# Danhong Injection Protects Hemorrhagic Brain by Increasing *Peroxiredoxin 1* in Aged Rats

**DOI:** 10.3389/fphar.2020.00346

**Published:** 2020-03-25

**Authors:** Shang Wang, Lie Yu, Guifang Sun, Yu Liu, Wentao Hu, Yanru Liu, Tao Peng, Xiaojun Wang, Jingliang Cheng, Aravintakumar Sr, Bo Qin, Hong Lu

**Affiliations:** ^1^Department of Neurology, The First Affiliated Hospital of Zhengzhou University, Zhengzhou, China; ^2^Department of Magnetic Resonance, The First Affiliated Hospital of Zhengzhou University, Zhengzhou, China; ^3^Translational Medicine Centre, The First Affiliated Hospital of Zhengzhou University, Zhengzhou, China

**Keywords:** aged rat, Danhong injection, intracerebral hemorrhage, hematoma volume, *Peroxiredoxin 1*

## Abstract

Intracerebral hemorrhage (ICH) is a severe cerebrovascular disease with a high incidence, mortality and disability rate. Danhong injection (DHI) is beneficial for ischemic stroke, but is prohibited for ICH due to risk of bleeding. The present study aims to explore the potential therapeutic time window and molecular mechanism of DHI in a collagenase-induced ICH model in aged rats. DHI administration after ICH could significantly improve body weight and neurological deficits, and reduce the hematoma volume and brain water content when compared to the vehicle control. Furthermore, the protective effect of DHI administration on days 1–3 after ICH was superior to those on days 3–5 or 7–9 after ICH. DHI remarkably increased the *Peroxiredoxin 1* (*Prx1*) expression in astrocytes and reduced the expression of inflammatory factors tumor necrosis factor-α (TNF-α) and interleukin-β (IL-1β) after ICH. The immediate treatment of *Prx1* inhibiter chelerythrine (Che) after ICH abolished the protective effect of DHI. Furthermore, the Che treatment reduced the expression of *Prx1* in astrocytes, but increased the expression of TNF-α and IL-1β after ICH. DHI treatment could not reverse these changes. Therefore, the earlier DHI is administered, the better the neuroprotective effect. DHI exerts antioxidative and anti-inflammatory function by increasing *Prx1* in astrocytes. These present results may change the established understanding of DHI, and reveal a novel treatment approach for ICH.

## Highlights

- DHI may be used to treat intracerebral hemorrhage.
- The earlier DHI is administered, the better the neuroprotective effect.
- DHI exerts antioxidative and anti-inflammatory function by increasing *Prx1* in astrocytes.

## Introduction

Intracerebral hemorrhage (ICH) is a catastrophic disease that causes severe disability and high mortality in adults. The 1-month mortality rate of ICH patients can reach up to 40%, although significant progress has been made in clinical treatment (Cordonnier et al., 2018; Hemphill et al., 2018). The cascading events induced by ICH are the main cause of secondary damage (Ironside et al., 2019). Oxidative stress and inﬂammation have been recognized as the major disruptive factors after ICH (Wang, 2010; Hu et al., 2016). Furthermore, these two are closely correlated. Oxidative stress can mediate the expression of proinﬂammatory cytokines such as tumor necrosis factor-α (TNF-α) and interleukin-β (IL-β), while proinﬂammatory cytokines can upregulate the production of reactive oxygen species (ROS). Therefore, approaches that could inhibit oxidative stress and/or inflammation can reduce hematoma volume and promote neurological recovery after ICH (Wang et al., 2018).

Danhong injection (DHI) is a traditional Chinese medicine extracted from two herbs *Salviae miltiorrhiza* Bunge (Danshen, China) and *Carthamus tinctorius* L (Honghua, China). The high-performance liquid chromatography (HPLC) analysis revealed that the main components of DHI are flavonoids and phenolic compounds, such as tanshinone, tanshinol acid, salvianolic acid B, protocatechuic aldehyde, rosmarinic acid, and hydroxysafflor yellow A (Liu et al., 2013; Li et al., 2017). The quality control of DHI is strictly according to the standard of the China Food and Drug Administration (CFDA), and fingerprint technology has been adopted in the process of production to ensure its quality (SFDA, 2002). DHI has been considered to accelerate blood circulation and remove blood stasis (Zhang et al., 2016). Hence, this has been widely used in Chinese clinical practice for treating cardiovascular and cerebral occlusive diseases (Chen et al., 2011), such as myocardial and cerebral ischemia injury, but is prohibited for ICH treatment according to its instruction for use (Guan et al., 2013; Guo et al., 2015). Its remarkable neuroprotective effects are mainly attributed to the antioxidative and anti-inflammatory function of DHI (Sun et al., 2009; Wang et al., 2016; Lyu et al., 2017). Therefore, the investigators explored whether DHI could be used to treat ICH due to its strong antioxidative and anti-inflammatory effects.

*Peroxiredoxins (Prxs)* is a ubiquitous family of antioxidant enzymes, which plays a dominant role in regulating the level of peroxides within cells and in protecting neurons from oxidative insult (Perkins et al., 2015). Recent studies have revealed the additional functions of *Prxs* in stress-induced gene expression and inflammation-related biological reactions, such as tissue repair and parasite infection. Notably, *Prx1* is the most abundant subtype in mammals. This belongs to the 2-Cys *Prxs* subfamily, which is a homodimer in cytosol and utilizes thioredoxin1 as an electron donor to directly convert hydrogen peroxide (H_2_O_2_) into H_2_O (Rhee et al., 2005; Ledgerwood et al., 2017). Mammalian sterile twenty (Mst)1, which is a serine/threonine protein kinase, can be activated by cellular stressors including H_2_O_2_, and Mst1 inactivates *Prx1* by phosphorylating it at Thr-90 and Thr-183 (Rawat et al., 2013). Chelerythrine (Che) is a special agonist for Mst1 (Yamamoto et al., 2003). Increasing the level of Mst1 can specifically induce the phosphorylation of *Prx1* leading to the inactivation of its biological function. The phosphorylation of *Prx1* could not be detected though the anti-*Prx1* antibody.

The present study attempted to explore the potential therapeutic time window and underlying mechanism of DHI for treating ICH. These results would provide a theoretical foundation and a novel strategy for ICH clinical treatment with DHI.

## Materials and Methods

### Animals

The present study was carried out according to the recommendations of the Institutional Animal Care and Use Committee, and approved by the Ethics Committee of Zhengzhou University. Adult male Sprague-Dawley (SD) rats, weighing 600–700 g (18 months old) (Masoro, 1980), were purchased from the Animal Center of Henan province, and subjected to ICH. Animals were individually fed and kept in cages at 22°C ± 2°C,with a relative humidity of 50% ± 10% and a 12-h light/dark cycle. A maximum of six rats were kept in a cage (470 × 300 × 150 mm^3^). These animals had free access to food and water.

### ICH Rat Model

The ICH model was carried out based on a previous approach (Rosenberg et al., 1990). Rats were fixed in a stereotactic frame (RWD Life Science, Shen Zhen, China) after being anesthetized with 10% chloral hydrate (intraperitoneal injection). Then, 1-mm craniectomy was performed and a stereo-tactical guided needle was inserted into the right striatum at the following coordinates relative to the bregma: 0.2 mm anterior, 3.0 mm lateral, and 6.0 mm deep. Then, 2 µl of collagenase VIIs (0.25 U/µl, Sigma-Aldrich) was injected at a stable speed of 0.2 µl/min. In order to prevent backflow, the needle was left in place for 10 min. Rats in the sham group received needle insertion, but without collagenase injection. After the injection, the needle was removed, the burr hole was filled with bone wax, and the wound was sutured.

### Magnetic Resonance Imaging

Based on a previous study (Del Bigio et al., 1996; Yang et al., 2017), the hematoma volumes were assessed after ICH (n = 12) by magnetic resonance imaging (MRI), which was conducted on a 3.0-T horizontal bore magnet MRI system (General Electric, USA). A birdcage volume resonator was used to attain the radiofrequency transmission, and the signal was received *via* a four-element surface coil located over the head of the rat. In order to accurately position the rat inside the magnet bore, gradient-echo pilot scans were conducted at the initiation of each imaging session. The T2-weighted images were acquired using 15 consecutive slices of 2-mm thickness. Then, the hematoma volumes were manually traced from the T2 maps, and the mean signal intensity was measured by two imaging analysts, who blinded to the experimental conditions. During the scanning process, each rat was covered with a quilt to maintain body temperature.

### Drug Administration

The DHI (drug approval number: Z20026866; product batch number: 13062020) was provided by BuChang Pharmaceutical Co. Ltd. (HeZe, Shandong, China), and prepared according to the statement of the CFDA. Briefly, Radix et Rhizoma Salviae Miltiorrhizae (750 g) was powdered and infused into 30% ethanol (7.5 L) for 1 h at 50°C for two times. Then, the extract was filtrated and 250 g of Flos Carthami was added into the residue. Afterwards, the mixture was immersed into 2.5 L of water for 1 h at 35°C for two times. The water extract was mixed with the alcoholic extract, and vacuum evaporated to a relative density of 1.10–1.20 (65°C). Then, in order to obtain the isotonic solution with a pH value of 6–7, the appropriate sodium chloride and sodium hydroxide for injection were added. The solution was filtered and stored at 4°C for 24 h. Afterwards, water was added for injection up to 1.0 L, and the liquid was filtered again, sterilized, and encapsulated into ampoules. Thus, the DHI was obtained (SFDA, 2002). The HPLC analysis revealed that the main components of the DHI were tanshinone, tanshinol acid, salvianolic acid B, protocatechuic aldehyde, rosmarinic acid, and hydroxysafflor yellow A (Jiang et al., 2015). The quality control standard for the DHI according to the National Drugs Surveillance Administrative Bureau is that the total amount of danshensu (molecular formula: C9H10O5) and protocatechuic aldehyde (molecular formula: C7H6O3) should not be lower than 0.5 mg in 1 ml of injection, as analyzed by HPLC (He et al., 2012).

**Figure 1:**
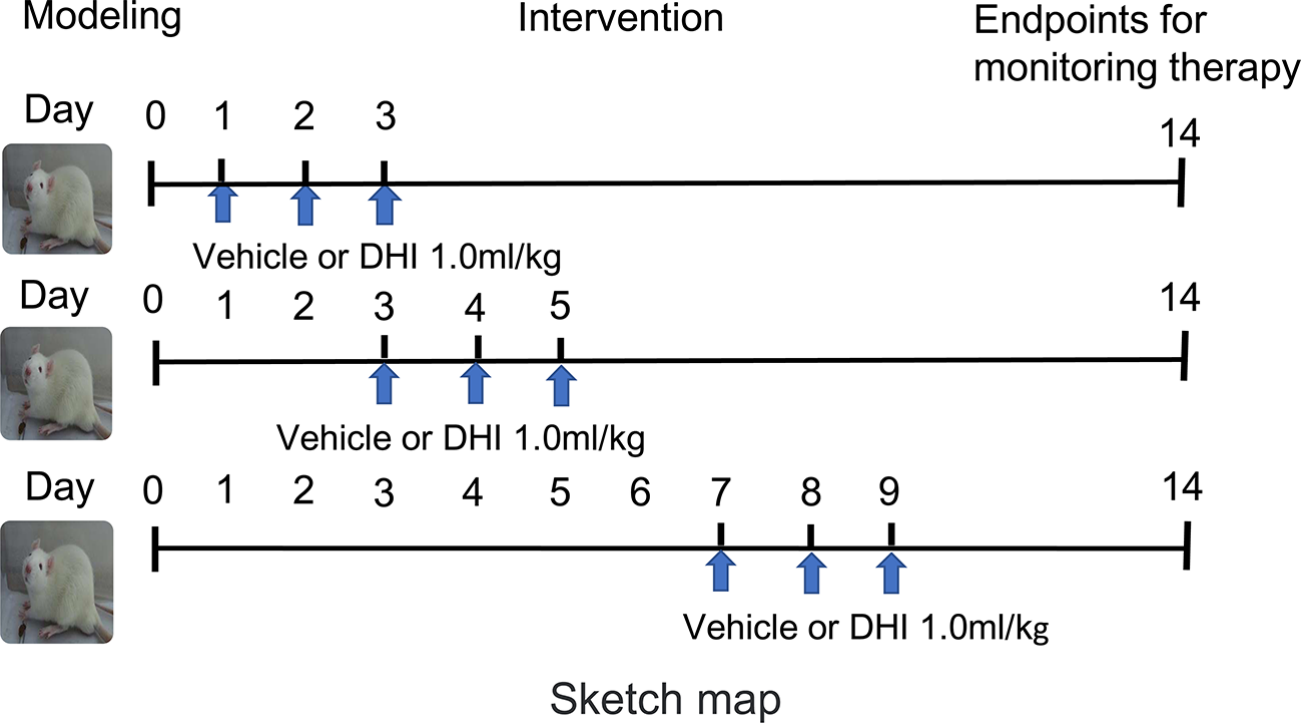
A sketch map of the experiment schedule for the study.

A commonly used dosage of DHI in ischemic stroke of a previous study was converted (Guo et al., 2015), and the clinical practice dose (1.0 ml/kg. d) was chosen for the present study. These animals were randomly divided into three groups (Han et al., 2019), as shown in **Figure 1**: (1) sham-operated (sham) group (n = 36), (2) ICH + vehicle group (n = 36), and (3) ICH + DHI group (n = 36). Animals in the ICH + DHI group were intraperitoneally administered with DHI on days 1, 3, and 7 after ICH (n = 12). For animals in the sham and ICH + vehicle group, equal volumes of saline were administered in the abdominal cavity.

In order to explore the underlying mechanism of DHI treatment in ICH, the MST1 agonist, Che was dissolved in dimethyl sulfoxide (DMSO, 10 µl, 1 mmol/L) (Sacchetti and Bielavska, 1998). Then, rats were further randomly divided into three groups: (4) ICH + Che group (n = 12), (5) ICH + DMSO + DHI group (n = 12), and (6) ICH + Che + DHI group (n = 12). Che was administered to the lesion core immediately after ICH, and DMSO was administered as the vehicle control. The DHI was continuously given at 1–3 days after ICH for rats in the ICH + DMSO + DHI and ICH + Che + DHI groups.

## Assessment of ICH Outcome

### Mortality, Body Weight, and Behavior Experiments After ICH

The body weight and neurologic deficits (through the 14-point modified neurological severity score (mNSS), corner turn test, and tape removal task) (Schaar et al., 2010; Lekic et al., 2012; Zhu et al., 2018) of rats were evaluated on days 1, 3, 7, and 14 after ICH. All behavior tests were performed in a blinded manner.

### Cresyl Violet Staining

At 14 days after ICH induction, each group of rats were given an MRI scan. Afterwards, rats (six rats in each group) were anesthetized and intracardially perfused with phosphate-buffered saline (pH 7.4), followed by 4% paraformaldehyde. Then, the brains were removed and immersed in 4% paraformaldehyde for 24 h, and were dehydrated with 30% sucrose solution for 3–5 days and at 4°C. Subsequently, the brain samples were frozen and cut into coronal frozen slices (thickness: 20 μm) using a cryostat microtome (Leica CM3050S-3-1-1, Bannockburn, IL). Then, the cryosections with cresyl violet to quantify the hematoma volume (Lan et al., 2017). The damaged areas were evaluated at a 10× objective using the Image-J software. The total injury volume in cubic millimeters was calculated as the sum of the damaged area multiplied by the distance between sections of 120 μm (Wu et al., 2017).

### Brain Water Content

At 14 days after the MRI scans in each group, rats were sacrificed to obtain fresh brain samples. The wet weight was obtained using a precision scale, and the samples were dried in an oven at a 100°C for 48 h. The brain water content was measured as a surrogate for brain edema (Han et al., 2016). This was calculated, as follows: [(wet weight − dry weight)/wet weight] ×100%.

## Immunofluorescence Staining

DHI was given at 24 h post-ICH as the following research target. Based on the established protocol (Lan et al., 2017), these sections were incubated overnight at 4°C with primary antibodies, including anti-*Prx1* (Abcam, ab59538 1:300), anti-NeuN (Abcam, ab177487 1:500), glial fibrillary acidic protein (GFAP) (Santa Cruz, sc33673 1:500), and anti-Iba-1 (Abcam, ab5076 1:500), followed by the appropriate fluorescence-conjugated secondary antibodies (Santa Cruz Biotechnology, CA, USA 1:300). Then, the sections were visualized using a fluorescence microscope (ZEISS Scope A1, ZEISS, Germany). Afterwards, the number of double-labeled cells in the striatum around the hematoma were counted (Chang et al., 2014). Brain sections with similar lesion areas were selected. Cell counts and the co-localization of *Prx1* with NeuN/GFAP/Iba-1 were analyzed using the Image-J software (1.4, NIH). Positive cells at 40 × 10 magnification from five optical fields in three sections per animal were averaged. The cell densities per square millimeter were calculated.

### Enzyme-Linked Immunosorbent Assay for Detection of Inflammatory Factors Levels

The concentrations of TNF-α and IL-6 in the brain homogenate solution of ICH rats was measured by enzyme-linked immunosorbent assay (ELISA) kits (USCN, Life Science Inc.), according to the manufacturer’s protocol.

### Statistical Analysis

All data were presented as mean ± standard deviation (SD), and SPSS 21.0 was used for all statistical analyses. First, all data were tested for normality of distribution with the Shapiro–Wilk test or Kolmogorov–Smirnov test. Then, the three groups comparison of different time points with normal distribution were compared using two-way analysis of variance (ANOVA), and the Mann–Whitney U test was used for nonparametric data. *P* < 0.05 was considered statistically significant.

## Results

### DHI Promotes Body Weight and Neurological Functional Recovery After ICH

**Figure 2:**
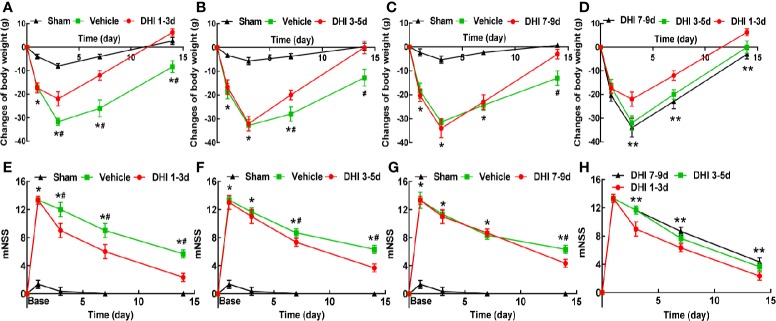
Danhong injection (DHI) treatment promotes the recovery of body weight and neurologic function. DHI treatment significantly promotes body weight recovery **(A–C)** and reduces modified neurologic severity score (mNSS, **E–G**) after intracerebral hemorrhage (ICH). **(D)** The administration of DHI on days 1–3 after ICH exhibits greater advantage in decreasing weight loss, when compared to the administration on days 3–5 or 7–9 (n = 12, *P* < 0.05). **(H)** DHI treatment on days 1–3 after ICH has a better effect on reducing neurological deficits, when compared to the treatment of DHI on days 3–5 or 7–9 (n = 12, *P* < 0.05). (*P < 0.05 *vs.* sham; **^#^***P* < 0.05 *vs.* vehicle, **P < 0.05 *vs.* DHI 3–5 and 7–9 days).

**Figure 3:**
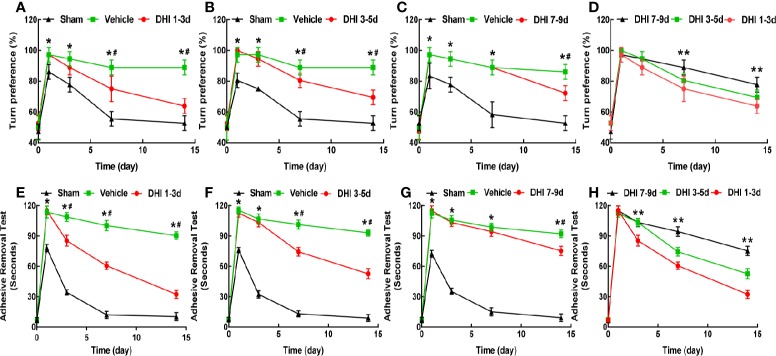
DHI treatment improves the turn bias and somatosensory deficits. DHI treatment significantly reduces the percentage of left turns in the corner turn test **(A–C)** and adhesive-removal time **(E–G)** after ICH. **(D)** The DHI administration on days 1–3 after ICH has a better effect on decreasing the percentage of left turns, when compared to the administration of DHI on days 3–5 or 7–9 (n = 6, *P* < 0.05). **(H)** The effect of DHI treatment on days 1–3 after ICH in reducing the adhesive-removal time was superior to the treatment performed on days 3–5 and 7–9 (n = 6, *P* < 0.05). (*P < 0.05 *vs.* sham; **^#^***P* < 0.05 *vs.* vehicle; **P < 0.05 *vs.* DHI 3–5 or 7–9 days).

Among the 211 rats, 11 rats died during the experiment. No death occurred in the sham group. The mortality rate in the vehicle and DHI treated group was 12.20% (5/41) and 14.29% (6/42), respectively. However, there was no difference in mortality between the vehicle-treated and DHI-treated groups. The body weight and neurological function of vehicle-treated rats significantly decreased during the first three days after ICH, when compared to sham operated rats (*P* < 0.001), and these gradually increased within 7–14 days after ICH (*P* < 0.005). Rats treated with DHI on days 1–3, 3–5, or 7–9 after ICH exhibited a significantly less body weight loss (**Figures 2A–C**), when compared to vehicle-treated rats (*P* < 0.05). The administration of DHI on days 1–3, 3–5, or 7–9 after ICH also significantly reduced the mNSS, (**Figures 2E–G**) the percentage of left turns in the corner turn test (**Figures 3A–C**), and the adhesive-removal time (**Figures 3E–G**), when compared to vehicle-treated rats. Furthermore, the protective effect of DHI treatment on days 1–3 after ICH on neurological function and body weight was superior, when compared to the treatment on days 3–5 or 7–9 (**Figures 2D, H** and **3D, H**; *P* < 0.05). In conclusion, the earlier the treatment of DHI, the better the ability to promote neurological function and body weight recovery.

### DHI Reduces Hematoma Volume and Brain Edema After ICH

**Figure 4:**
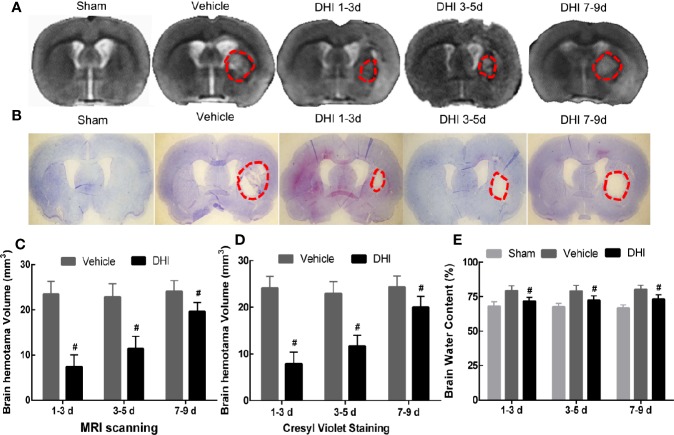
DHI treatment reduces hematoma volume and brain water content after ICH. The **(A, C)** Magnetic resonance imaging (MRI) scanning and **(B, D)** cresyl violet staining results revealed that DHI treatment significantly reduced hematoma volume, when compared with the control (n = 12, *P* < 0.01), and the hematoma volume in rats treated with DHI on days 1–3 after ICH was significantly lesser, when compared to that in the other two time point groups (n = 12, *P* < 0.01). **(E)** The brain water content test revealed that the DHI treatment reduced the brain water content, when compared to the vehicle treatment (n = 6, *P* < 0.05). However, the time of treatment did not affect the effect of DHI in reducing encephaledema (n = 6, *P* > 0.05). (**^#^***P* < 0.05 *vs.* vehicle).

The therapeutic effect of DHI on day 14 after ICH was verified. First, MRI scanning (**Figure 4A**) and cresyl violet (**Figure 4B**) staining were performed to assess the hematoma volume. The hematoma volume in the DHI group (1.0 mg/kg. d) significantly decreased, when compared to the vehicle group (**Figures 4C, D**; *P* < 0.01). However, rats treated with DHI on days 1–3 after ICH had the smaller cerebral hematoma size, when compared to rats treated with DHI, on days 3–5 or 7–9 (*P* < 0.05). Furthermore, the hematoma volume of rats treated with DHI on days 3–5 after ICH was smaller, when compared to rats treated with DHI on days 7–9 (**Figures 4C, D**). In addition, the DHI treatment simultaneously reduced the brain water content on day 14 at post-ICH (**Figure 4E**, n = 6; DHI at days 1–3, 72.64% ± 1.82%; DHI at days 3–5, 74.44% ± 1.65%; DHI at days 7–9, 75.02% ± 1.06%; vehicle, 78.31% ± 1.63%; *P* < 0.05). However, the time of treatment of DHI did not affect the reduction in encephaledema (**Figure 4E**, *P* > 0.05).

### DHI Treatment Upregulates Prx1 Expression After ICH

**Figure 5:**
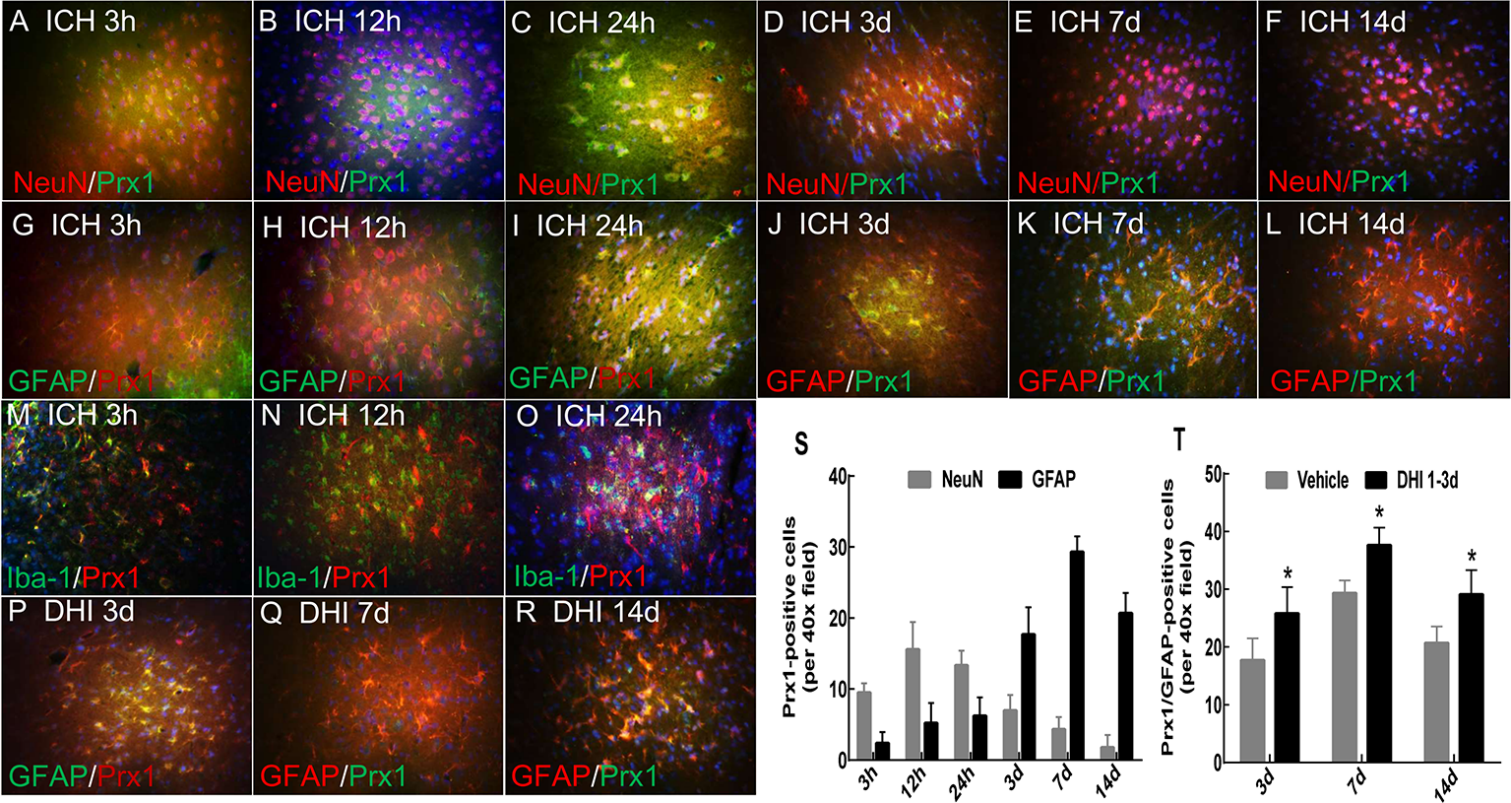
DHI upregulates the *Peroxiredoxin 1* (*Prx1*) expression in astrocytes after ICH. **(A–L)** The immunofluorescence staining results revealed that *Prx1* was mainly expressed in the cytoplasm of neurons within 24 h after ICH, peaked at 12 h, and gradually decreased **(A–F)**. The expression of *Prx1* in astrocytes was dramatically increased and peaked on day 7 after ICH **(G–L)**. **(M–O)** However, no *Prx1* was observed in Iba-1^+^ cells. **(P–R)** The DHI treatment on days 1–3 after ICH remarkably increased the expression of *Prx1* in astrocytes. Majority of *Prx1* positive cells were astrocytes. **(S)** The quantitation of *Prx1* expression in neurons and astrocytes after ICH. **(T)** The quantitation the effect of DHI treatment on days 1–3 after ICH on the *Prx1* expression in astrocytes (n = 6, *P* < 0.05). (Images are shown at 400× magnification, *P < 0.05 *vs.* vehicle).

Few studies have investigated the expression of *Prx1* after ICH. Therefore, the expression pattern of *Prx1* after ICH was identified. The immunofluorescence staining results revealed that the expression of *Prx1* was upregulated and peaked on day 7, and began to gradually decrease on day 14 after ICH (**Figures 5A–L, S**). In order to verify the source of *Prx1* after ICH, the expression of *Prx1* in astrocytes (GFAP+ cells), microglias (Iba-1+ cells), and neurons (NeuN+ cells) were assessed. The immunofluorescence staining results revealed that *Prx1* was mainly expressed in the cytoplasm of neuron within 24 h after ICH, and this gradually disappeared on day 3 (**Figures 5A–F, S**). The expression of *Prx1* in astrocytes was less than that in neuron within 24 h. This dramatically increased on day 3 and peaked on day 7 after ICH (**Figures 5G–L, S**). However, no *Prx1* was observed to express in Iba-1+ cells (**Figures 5M–O**). Since the neurological protective effect of the injection of DHI on days 1–3 after ICH was superior to that on days 3–5 and 7–9, the influence of DHI treatment on days 1–3 after ICH on the expression of *Prx1* was tested. The immunofluorescence staining results demonstrated that *Prx1* was mainly expressed in astrocytes on day 3 after the DHI treatment. The DHI treatment remarkably increased the expression of *Prx1* in astrocytes on days 3–14 after ICH, when compared to the vehicle treatment (**Figures 5P–R, T**; *P* < 0.05). These results indicate that DHI mainly affects *Prx1* expression in astrocytes rather than neurons.

### Che Inhibits the Effects of DHI on ICH by Inactivating Prx1

**Figure 6:**
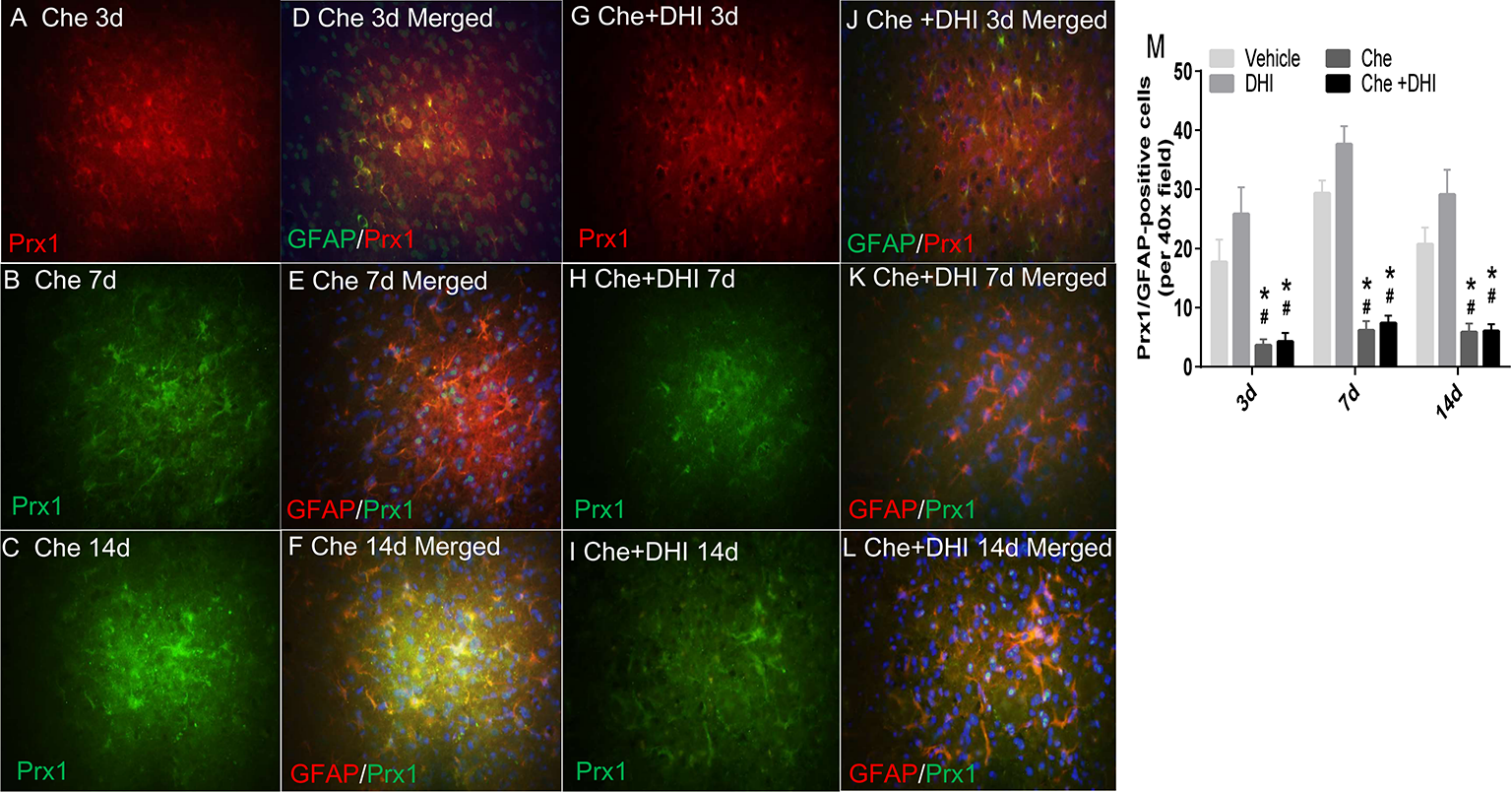
Che inhibits the expression of *Prx1* in astrocytes after ICH. **(A–F)** The effect of Che injection on *Prx1* expression on days 3 **(A, D)**, 7 **(B, E)**, and 14 **(C, F)** after ICH. **(G–L)** The effect of Che + DHI treatment on *Prx1* expression on days 3 **(G, J)**, 7 **(H, K)**, and 14 **(I, L)** after ICH. **(M)** The quantitative analysis results revealed that the immediate treatment of Che after collagenase injection significantly inhibited the expression of *Prx1* in astrocytes. DHI administration could not reverse the phenomenon. (Images are shown at 400× magnification; Che, chelerythrine; *P < 0.05 *vs.* vehicle, **^#^***P* < 0.05 *vs.* DHI).

**Figure 7:**
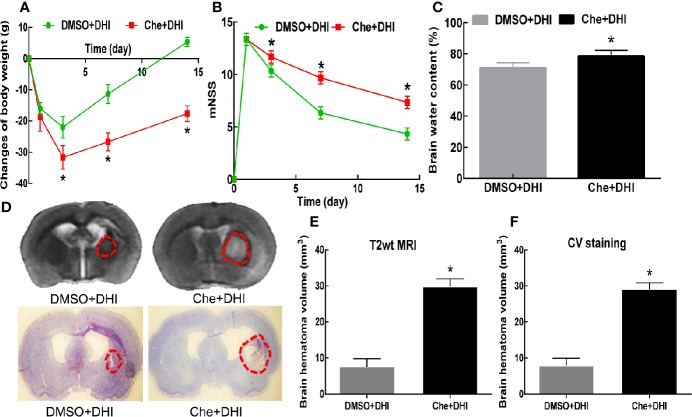
The inactivity of *Prx1* with Che after ICH abolishes the neuroprotective function of DHI. Che treatment impeded the recovery of body weight (**A**, n = 12) and neurological function (**B**, n = 12), and prevented the reduction in brain water content (**C**, n = 6) and hematoma volume (**D/E/F**, n = 6). [*P < 0.05 *vs.* dimethyl sulfoxide (DMSO) + DHI].

Based on the above findings, it was hypothesized that DHI exerts its neurological protective effect by upregulating the expression of *Prx1*. Che (a *Prx1* inhibitor) could specifically mediate the phosphorylation of *Prx1*, which leads to the inactivation of its biological function. It was found that the expression of *Prx1* in the ICH + Che + DHI and ICH + Che groups significantly decreased, when compared to the ICH group and ICH + DHI group (**Figures 6A–M**, *P* < 0.01). Furthermore, there was no difference in the expression of *Prx1* between the ICH + Che + DHI and ICH + Che groups on days 3, 7, and 14 after ICH (**Figures 6A–M**, *P* > 0.05). In addition, the inhibition of the biological function of *Prx1* using Che before the DHI injection prevented the recovery of body weight and neurological function, and the reduction of brain water content and hematoma volume, when compared to the DHI + DMSO treatment (**Figures 7A–E**, *P* < 0.05).

### DHI Inhibits the Expression of Inflammatory Factors After ICH

**Figure 8:**
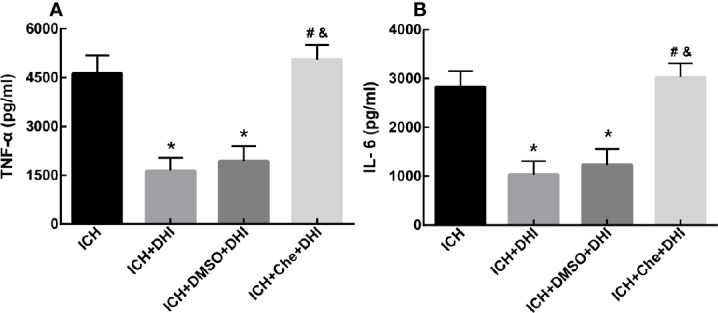
DHI reduces the expression of inflammatory factors tumor necrosis factor-α (TNF-α) and interleukin-6 (IL-6) after ICH. The levels of TNF-α and IL-6 significantly decreased in the ICH + DHI and ICH + DMSO + DHI groups, when compared to the vehicle group (**A, B**, n = 6). (*P < 0.05 *vs.* ICH). The expression of TNF-α and IL-6 was no longer inhibited by DHI after the injection of Che in the rat model of ICH (**A, B**, n = 6). (**^#^***P* < 0.05 *vs.* ICH + DMSO + DHI; ^&^*P* < 0.05 *vs.* ICH + DHI).

It has been reported that DHI could attenuate inflammatory reactions after ICH. Hence, the changes in TNF-α and IL-6 were detected. The ELISA results revealed that DHI treatment significantly inhibited the expression of TNF-α and IL-6 in ICH aged rats, when compared to the vehicle treatment (**Figures 8A, B**, *P* < 0.05). However, with the treatment of Che after ICH, the administration of DHI could no longer decrease the expression of TNF-α and IL-6 (**Figures 8A, B**, *P* < 0.05).

## Discussion

The collagenase-induced ICH model of aging rats in the present study simulated the clinical phenomenon of spontaneous ICH, which mainly occurs in aging adults. The present study is the first to demonstrate that DHI can be used to treat ICH, and that this has an outstanding neuroprotective effect on ICH. Furthermore, the earlier administration of DHI led to its better ability to promote neurological function and hematoma recovery. The molecular mechanism of the neuroprotective effect of DHI involved the upregulation of *Prx1* and enhanced its antioxidative and anti-inflammatory functions in astrocyte. These novel findings also suggested that *Prx1* is a potential target to ameliorate secondary brain injury and improve long-term neurologic recovery after ICH.

A previous study revealed that aging exacerbates the astroglial reaction in response to excitotoxic damage (Castillo-Ruiz et al., 2007), increases oxidative stress, and deteriorates neurological function due to loss of neurotransmission (Monti et al., 2004). Activated glia presents with different changes in different regions in aged brain including age-related hypertrophy in the frontal cortex and a numeric increase in the hippocampus (Amenta et al., 1998). Astrocytes in aged rats also exhibit a region-specific regulation function, which can attenuate the injury-induced cytokine response after excitotoxic damage (Campuzano et al., 2009). Given these afore-mentioned findings, it can be concluded that aged rats are different from young and adult rats when these are exposed to insults. These differences are reflected not only in its self-defense function, but also in the process of damage (Zhao et al., 2015). Thus, aged rats were chosen as the present experimental objects, in order to explore the role of DHI and its potential molecular mechanism in ICH.

ICH is a devastating disease that brings serious burden to humans. Most patients with ICH would retain varying degrees of disability. However, drugs for treating ICH are very limited and have poor efficacy (Wang et al., 2018). DHI is a neuroprotective agent (Qian et al., 2018), and is widely used to treat cerebral (Wang et al., 2016) and myocardial ischemic diseases (Guan et al., 2013). Traditional theory deems that DHI can accelerate blood circulation and remove blood stasis. To date, it is still prohibited to use this to treat ICH due to the high risk of hemorrhage. After careful analysis of the literature regarding DHI, it was found that the most important mechanism of DHI treatment for ischemic diseases are the antioxidative stress and anti-inflammatory function. The main causes that lead to the progression of ICH are also oxidative stress and inflammation (Li et al., 2017; Feng et al., 2019). Furthermore, a previous clinical study revealed the protective effect of DHI for treating traumatic intracranial hematoma (Sun et al., 2009). Therefore, an attempt was made to explore the efficacy of DHI in treating ICH in different time windows. These above results illustrate that DHI has a neuroprotective effect for ICH. First, this did not increase motility. Second, DHI promoted the recovery of body weight and neurological function. Third, this reduced brain hemorrhagic volume and brain water content, when compared to the vehicle. Next, the dissimilitude of the protective effect of DHI treatment at different time points was assessed. It was found that there were remarkably different efficiencies among the three groups. The neuroprotective effect of DHI treatment at days 1–3 was better, when compared to the treatment on days 3–5 or 7–9, regardless of the recovery of weight, neurological function, or reduction of hematoma and brain edema. In conclusion, DHI can be used to treat ICH, even in the acute phase.

A study proposed that DHI can enhance the antioxidant capacity of micro-vascular endothelial cells in the context of cerebral hypoxia (Lyu et al., 2017). In addition, DHI exerts its biological effects by changing the Nrf2 levels and upregulating the level of SOD, GSH, and MDA after ischemic stroke (Guo et al., 2014). Furthermore, the fractions of 5–7 and 17–19 in a ternary network have been demonstrated to be the main active components of DHI (Wang et al., 2016). *Prx1* is an antioxidative stress protein. However, its biological activity is inhibited by heme after ICH. Furthermore, the antioxidative function of *Prx1* relays on the co-expression of HO-1 (Nakaso et al., 2000; Zhang et al., 2017). However, these studies do not depict the dynamic changes of *Prx1* after ICH. It was considered that scavenging H_2_O_2_ may be the most effective approach of DHI in exerting neuroprotection after ICH. *Prxs* have been shown to be the most effective protein to scavenge H_2_O_2_, especially *Prx1* (Mizusawa et al., 2000). Recent evidence has indicated that *Prx1/2* protects the brain against H_2_O_2_-induced apoptosis after subarachnoid hemorrhage (Lu et al., 2019). Another study depicted that antioxidant *Prx1* is more highly expressed than other antioxidant enzymes in monocytes and macrophages, and that *Prx1* deficiency leads to excessive oxidative stress and impairs the maintenance of autophagic flux in macrophages (Jeong et al., 2018). Thus, *Prx1* was chosen to further determine the effect of DHI. The dynamic changes of *Prx1* after ICH were first presented in the present study. It was found that *Prx1* was upregulated and peaked at 12 h in neuron, and on day 7 in astrocytes after ICH. A study that conducted a proteomic analysis revealed that *Prx1* was downregulated in peroxide, while the catalase expression was upregulated at 3 h after ICH (Ren et al., 2014). Another study concluded that the expression level of *Prx1* in the hematoma region was higher than that in other areas at 1 day after blood injection (Nakaso et al., 2000). This discrepancy may be due to the difference of the experimental objects and methods of study. Immunofluorescence staining was performed to determine the expression level and cellular location. Published literatures have focused on the integrated level of *Prx1* in a whole hemorrhagic brain using the proteomic analysis method. The present results demonstrate that the expression of *Prx1* was further elevated by DHI especially on days 3 and 7. The revelation of the dynamic changes of *Prx1* after ICH lays the foundation for further study.

Next, efforts were made to observe the cellular position of *Prx1*. A study revealed that *Prx1* was mainly expressed in astrocytes, and elevated after subarachnoid hemorrhage (Lu et al., 2019). Nakaso et al. elaborated that HO-1 and *Prx1* were induced in reactive astrocytes (mainly at days 14 and 28) around the hemorrhagic region. However, both proteins were not induced in neurons (Nakaso et al., 2000). Another research revealed that *Prx1* was a major hemorrhagic stress-inducible isoform of *Prxs* in ICH. The insult stimulated the *Prx1* expression and mediated extracellular release, leading to the activation of TLR-4/NF-κB signaling and the production of inflammatory cytokines (TNF-α, IL-6, and IL-17) (Liu et al., 2016). The present study revealed that *Prx1 was* mainly expressed in neuronal cells (which peaked at 12 h) in the peri-hematoma region in the first 3 days after ICH. Simultaneously, *Prx1* increased in astrocytes at the beginning of the ICH, peaked on day 7, and subsequently decreased on day 14. No obvious *Prx1* was detected outside the astrocyte. In contrast, the expression of *Prx1* was further elevated in astrocytes, and the expression of inflammatory factors TNF-α and IL-6 decreased after treatment with DHI. From these results, it could be concluded that DHI intracellularly promotes the expression of *Prx1* after ICH and exerts its neuroprotection though its anti-inflammatory effect. Furthermore, in order to further explore the neuroprotective effect of DHI, Che, an indirect inhibitor of *Prx1*, was chosen to further verify the role of *Prx1* in ICH. Che promotes the phosphorylation of *Prx1*, which results in the loss of its antioxidant and anti-inflammation effects. These changes lead to the expansion of the hematoma volume, and deterioration in neurological function. DHI treatment cannot reverse the detriments caused by the inactivation of *Prx1*. Taken together, the present results demonstrate the vital role of *Prx1*, and that DHI exerts its neuroprotective effect through the upregulation of *Prx1*.

A previous study indicated that *Prx1* appeared to act as a sensitive biomarker to ROS (e.g., H_2_O_2_) and represented an initial response to stress to maintain redox homeostasis, preventing oxidative damage to lipids and proteins (Scotton et al., 2020). In addition, *Prx1* negatively regulated TLR4 signaling for NF-κB activation by inhibiting TRAF6 ubiquitin-ligase activity (Min et al., 2018). Furthermore, ROS can activate inflammatory pathways that involve NF-κB signaling, and immune system activation. The production of ROS was orchestrated by inflammatory transcription factors, including nuclear factors derived from erythroid 2 (Nrf2) and NF-κB (Bakunina et al., 2015). These involved evidences showed that inflammation and oxidative stress are closely correlated. Oxidative stress can mediate inflammation, while inflammation causes damage through oxidative stress. Therefore, it can be concluded that DHI exerts its neuroprotective effect though antioxidative and anti-inflammatory functions.

In summary, the present study verified the efficacy of DHI in the treatment of ICH in the acute phase. DHI exhibited its antioxidative and anti-inflammatory mechanism by upregulating the *Prx1* expression in neurons and astrocytes in a collagenase-induced ICH aged rat model. However, the present study has limitations. One limitation is that merely aged male rats were involved, and the collagenase-induced ICH aged male rat model could not fully simulate the clinical ICH. Another limitation is that DHI is a traditional Chinese medicine, and its ingredients are not fully elucidated. Further studies are needed to differentiate the effective component. Overall, with multiple cellular and molecular targets, DHI exerts its impact on preclinical investigation, and holds therapeutic promise for patients with ICH.

## Data Availability Statement

The datasets generated for this study are available on request to the corresponding authors.

## Ethics Statement

The animal study adhered to the institutional guidelines of the Animal Care and Use Committee of Zhengzhou University.

## Author Contributions

SW and LY contributed equally to this work, both designed and performed these experiments, drafted and revised the manuscript. HL designed, and supervised the research, and revised the manuscript. GS, YuL, WH, YaL and TP, XW, AS and BQ supervised the research and revised the manuscript. JC provided the magnetic resonance instrument. All authors approved the manuscript.

## Funding

This study was supported by grants from the National Natural Science Foundation of China (No. 81971175), the Henan Medical Science and Technology Key Project (Nos. 2018020050 and 2018020094), the Henan Key Special Project for Development and Extension (No. 202102310076), and the Henan Provincial Higher Education Key Research Project Plan (No. 18A320052).

## Conflict of Interest

The authors declare that the research was conducted in the absence of any commercial or financial relationships that could be construed as a potential conflict of interest.

## Abbreviations

ICH, intracerebral hemorrhage; DHI, Danhong injection; *Prx1, Peroxiredoxin 1*; Che, chelerythrine; mNSS, modified neurologic severity score; PBS, phosphate-buffered saline; MRI, magnetic resonance imaging; H_2_O_2_, hydrogen peroxide; Mst, mammalian sterile twenty; GFAP, glial fibrillary acidic protein.
